# Deficient early canonical BMP9-SMAD signaling and dysregulated macrophage microenvironment characterize the failure of bone healing in a critical-size defect model

**DOI:** 10.3389/fendo.2026.1815554

**Published:** 2026-07-20

**Authors:** Kai Zhong, Yufei Shao, Yu Zhou, Zengyi Huang, Xing Liu

**Affiliations:** 1 Department of Orthopedics, Children’s Hospital of Chongqing Medical University, National Clinical Research Center for Children and Adolescents’ Health and Diseases, Ministry of Education Key Laboratory of Child Development and Disorders, Chongqing, China; 2 Functional Validation Platform for Pathogenic Genes in Pediatric Rare Diseases, Children’s Hospital of Chongqing Medical University, National Clinical Research Center for Children and Adolescents’ Health and Diseases, Ministry of Education Key Laboratory of Child Development and Disorders, Chongqing, China; 3 Department of Radiology Children’s Hospital of Chongqing Medical University, National Clinical Research Center for Children and Adolescents’ Health and Diseases, Ministry of Education Key Laboratory of Child Development and Disorders, Chongqing, China; 4 Jiangxi Hospital Affiliated to Children’s Hospital of Chongqing Medical University, Nanchang, China

**Keywords:** atrophic non-union, BMP9, macrophage polarization, osteoclast, SMAD signaling

## Abstract

**Introduction:**

Atrophic non-union is a significant clinical challenge associated with impaired early inflammation resolution and delayed osteogenesis. Although the osteoimmune microenvironment is critical for bone healing, the specific molecular mechanisms regulating this process require further elucidation.

**Methods:**

We utilized a 6-mm critical-size rat femoral defect model and time-series RNA sequencing to explore potential molecular mechanisms. Additionally, primary mouse bone marrow-derived macrophages (BMMs) were used for in vitro validation of in vivo findings.

**Results:**

Histological and transcriptomic analyses identified 1-2 weeks post-fracture as a critical window characterized by significant inhibition of the canonical BMP9-SMAD signaling cascade in the non-union microenvironment. *In vivo*, this defect was associated with sustained accumulation of M1 macrophages, impaired reparative M2 polarization, and osteoclast-mediated bone resorption. *In vitro* studies further validated the in vivo results, confirming that BMP9 promotes M2 macrophage polarization, upregulates chemokines Ccl2 and Ccl7, and inhibits RANKL-induced osteoclastogenesis through the canonical SMAD-ID1 pathway.

**Discussion:**

Early loss of canonical BMP9-SMAD signaling disrupts the balance between immune cell recruitment and bone remodeling, contributing to the development of atrophic non-union in critical-size defects. Targeted modulation of the BMP9-driven osteoimmune axis may offer a potential therapeutic strategy for impaired bone healing.

## Introduction

Fracture healing is a highly coordinated physiological process that requires precise spatiotemporal interactions among diverse cell lineages, growth factors, and the extracellular matrix (1). Although most fractures heal successfully, approximately 10% to 15% of patients experience delayed healing or non-union, which imposes a substantial burden on both the patients and the healthcare system (2–4). Based on the traditional Weber-Cech classification, non-unions are broadly categorized into hypertrophic and atrophic types (5). Atrophic non-union is particularly challenging to treat, as it is characterized by a severe lack of biological osteogenic potential, minimal to no callus formation, and progressive fibrosis at the fracture ends (2). Historically, the primary etiology of atrophic non-union was attributed to inadequate local vascularization (6). However, recent histological analyses have challenged this paradigm, revealing that vascular density in certain human and murine atrophic non-union tissues is comparable to that of normal healing fractures, and that the non-union tissue is not necessarily avascular (7, 8). Consequently, the research focus has increasingly shifted toward the critical early cellular activities and the local osteoimmune microenvironment as the potential factors determining fracture fate (9).

In the nascent phase of fracture repair, the fracture hematoma serves as a temporary scaffold and a rich reservoir of immune cells that initiate the inflammatory cascade (10). Within this osteoimmune microenvironment, macrophages exhibit remarkable plasticity and exist along a functional continuum, primarily simplified into pro-inflammatory (M1) and pro-reparative (M2) phenotypes (11, 12). A successful healing cascade requires a timely and orchestrated transition from an M1-dominant phase to an M2-dominant phase, which promotes angiogenesis, extracellular matrix synthesis, and osteoblast differentiation (13). An imbalance in this M1/M2 ratio leads to sustained local inflammation, which significantly blunts endochondral ossification and fosters a fibrotic non-union state (14). Furthermore, macrophages and bone-resorbing osteoclasts share a common myeloid progenitor origin, representing two competing differentiation pathways (11). Sustained inflammatory signaling not only impairs M2 polarization but can also persistently stimulate osteoclastogenesis, further shifting the local balance toward pathological bone resorption (15, 16). Crucially, in rodent models, this immune transition and the subsequent initiation of the osteogenic cascade must occur within a narrow timeframe, typically the first 1 to 2 weeks post-injury. Failure to resolve inflammation within this critical timeframe inevitably leads the fracture to become a non-union (10, 17).

To investigate the molecular mechanisms underlying this early microenvironmental failure, establishing a reliable and reproducible animal model is imperative. In this study, we employed a modified rat femoral critical-size defect (CSD) model, characterized by a 6-mm diaphyseal defect stabilized by a metal clip (18). We acknowledge that a 6-mm defect in a rat femur represents a critical-size defect that surpasses the inherent biological capacity for spontaneous bridging (19). However, this extreme physical condition makes it a valuable tool for amplifying the pathological process of early healing failure. The tissue at the defect margin recreates the biological characteristics of atrophic non-union, such as restricted bone formation, persistent fibrous tissue proliferation, and persistent inflammation, thus providing an ideal “magnified window” for exploring the underlying mechanisms leading to non-union.

Among the many factors regulating bone repair, bone morphogenetic protein 9 (BMP9) has been identified as one of the most potent osteogenic inducing factors (20). However, its specific regulatory role within the early osteoimmune microenvironment, particularly concerning macrophage polarization and osteoclastogenesis, remains a subject of intense academic debate. On one hand, *in vitro* studies utilizing immortalized macrophage cell lines (e.g., RAW 264.7 cells) have suggested that BMP9 may promote osteoclast differentiation via non-canonical ALK1 and ERK1/2 signaling pathways (21). On the other hand, recent studies employing primary bone marrow-derived macrophages (BMMs) and *in vivo* ovariectomized mouse models have demonstrated that BMP9 strongly inhibits RANKL-induced osteoclastogenesis through the canonical SMAD pathway, thereby exerting a protective, anti-resorptive effect (22). We hypothesize that these contradictory findings stem from the inherent heterogeneity of macrophage populations and their context-dependent responses to local microenvironmental cues (23).

Based on these premises, the present study aims to elucidate the intricate cross-talk between BMP9 signaling and the innate immune network during the critical early window of fracture repair.

## Materials and methods

### Animals

All animal experiments were designed and reported in accordance with the ARRIVE 2.0 guidelines and approved by the Animal Ethics Committee of Chongqing Medical University Children’s Hospital (CHCMMU-IACUC20251105003). 12-week-old male Sprague-Dawley rats (weighing 300–350 g) and 4-week-old male C57BL6/J mice were maintained under specific pathogen-free conditions with free access to food and water and a 12-hour light cycle. For the *in vivo* study, the rats were randomly assigned to either the Fracture group or the Non-union (Critical-Size Defect) group.

### Surgical procedure

To establish a rat model of non-union, we adapted Garcia’s surgical technique with modifications tailored to create a critical-size defect (8). All surgical procedures were performed under standard aseptic conditions. Anesthesia was induced by inhalation of 2.5% isoflurane (RWD, China), followed by maintenance with 2% isoflurane and oxygen via a face mask. The right hindlimb was shaved and prepared aseptically. The right femur was exposed via parapatellar and lateral approaches. A custom-made 10-millimeter metal clamp made of medical-grade stainless steel was implanted into the femur from the dorsal to the ventral side. Under continuous saline perfusion, a 6-millimeter defect (more than twice the diameter of the mid-femur) was created using a reciprocating saw, thereby effectively establishing a critical-size defect. A 0.8-millimeter-diameter intramedullary K-wire was then inserted into the medial side of the pre-placed metal clip. The two components provided axial and selective stability through a press-fit mechanism. The standard Fracture group underwent a similar procedure with simple transverse osteotomy, but without the removal of the bone column or the placement of the metal clip. Postoperatively, carprofen (5 mg/kg) was administered subcutaneously for pain relief for 3 consecutive days.

### X-ray analysis

Postoperative radiographs of the operated femur were taken under anesthesia at 2, 5, 10, and 15 weeks post-surgery (n = 6 per group/time point) to assess bone healing.

### Antibodies and reagents

All primary and secondary antibodies utilized in this study were exclusively purchased from Affinity Biosciences (Changzhou, China). Primary antibodies included: anti-BMP9 (DF7758, 1:200 for IHC, 1:1000 for WB), anti-Phospho-Smad1/5/9 (AF8313, 1:100 for IHC, 1:1000 for WB), anti-ALP (DF12525, 1:100 for IHC), anti-CD86 (DF6332, 1:200 for IF), anti-CD206 (DF4149, 1:200 for IF), anti-F4/80 (DF4874, 1:200 for IF), and anti-GAPDH (AF7021, 1:5000 for WB).

### Histology, immunohistochemistry, and histomorphometry

The right femur was fixed in 4% paraformaldehyde at 4 °C for 24 hours, followed by decalcification in 10% EDTA solution for 6 weeks (solution replaced every 3 days). After decalcification and removal of the implant, the tissue is embedded in paraffin. To avoid spatial heterogeneity and selection bias, the specimens were divided into two along the midline. Starting from this surface, sections were cut of 30 μm intervals to obtain 15 consecutive representative slices for each animal. Slices were randomly selected for subsequent operations. Sections were stained with hematoxylin-eosin (H&E) and Masson’s trichrome. For immunohistochemistry (IHC), sections underwent antigen retrieval using Tris-EDTA buffer (pH=9) or citrate buffer (pH=6), followed by 3% hydrogen peroxide quenching and normal serum blocking. Sections were incubated with primary antibodies (BMP9, p-Smad1/5/9, ALP) overnight at 4 °C, followed by HRP-conjugated secondary antibodies and DAB development. Immunofluorescence (IF) staining was performed using a mixture of anti-CD86 or anti-CD206 with anti-F4/80, followed by Alexa Fluor^®^ 568/488 secondary antibodies. Tartrate-resistant acid phosphatase (TRAP) staining was performed to evaluate osteoclast activity. In accordance with the American Society for Bone and Mineral Research (ASBMR) guidelines, mature osteoclasts were strictly defined as TRAP-positive multinucleated cells containing three or more nuclei and firmly attached to the bone surface. Quantitative histomorphometry was performed using ImageJ software across 5 random high-power fields per slide, and the osteoclast number was normalized to the bone surface (N.Oc/BS).

### Quantitative real-time PCR analysis

Total RNA was extracted from rat callus tissue and mouse bone marrow matrix using TRIzol reagent (Invitrogen, USA). Specifically, for the non-union group, the boundary for RNA extraction was defined as 6mm defect gap plus a 2mm edge adjacent to the host bone at both ends. Correspondingly, for the fracture group, 5mm segments were taken at each end centered on the fracture line. The concentration and purity of RNA were evaluated using a NanoDrop spectrophotometer. For cDNA synthesis, 1 μ g of total RNA was reverse transcribed into cDNA using PrimeScript RT kit (Takara, Japan). Real time PCR was performed using SYBR Green Master Mix (Vazyme, China) on CFX96 Touch real-time PCR detection system (Bio Rad, USA). The relative mRNA expression levels were calculated using the 2^-ΔΔCt^ method, with *Gapdh* serving as the internal reference gene. Primer sequences are listed in **Table 1**.

**Table 1:** Primer sequence of qPCR.

Species	Gene	Primer sequence (5’→3’)
Rat	*Gapdh*	F: ACGGCAAGTTCAACGGCACAG; R: CGACATACTCAGCACCAGCATCAC
	*Ocn*	F: GGACCCTCTCTCTGCTCACTCTG; R: ACCTTACTGCCCTCCTGCTTGG
	*Opg*	F: TCCCTTGCCCTGACTACTCTTATAC; R: CCTTCCTCACATTCGCACACTC
	*Nfatc1*	F: AGGGTGCGGCTGGTCTTCC; R: GCTGTCTGTGCTCTGCTTCTCC
	*Sost*	F: CAAGCCTTCAAGAATGATGCCACAG; R: CCGGTTCATGGTCTGGTTGTTCTC
	*Runx2*	F: CTTCGTCAGCGTCCTATCAGTTCC; R: TCCATCAGCGTCAACACCATCATTC
Mouse	*Gapdh*	F: AGGTCGGTGTGAACGGATTTG; R: GGGGTCGTTGATGGCAACA
	*Nos2*	F: GTTCTCAGCCCAACAATACAAGA; R: GTGGACGGGTCGATGTCAC
	*Nfatc1*	F: GGAGAGTCCGAGAATCGAGAT; R: TTGCAGCTAGGAAGTACGTCT
	*c-Fos*	F: CGGGTTTCAACGCCGACTA; R: TGGCACTAGAGACGGACAGAT
	*Cd206*	F: CTCTGTTCAGCTATTGGACGC; R: TGGCACTCCCAAACATAATTTGA
	*Bmp9*	F: CCCTGGGATTGTCTGGAGC; R: AGGTTAAGGCTGCGTAGGAAA
	*Cd86*	F: TCAATGGGACTGCATATCTGCC; R: GCCAAAATACTACCAGCTCACT

### RNA sequencing and bioinformatics analysis

Total RNA was extracted from tissues and cells using TRIzol (Invitrogen, USA). We used three independent biological replicates (N = 3) per group. This included *in vivo* rat callus tissues (weeks 1, 2, 5, and 15) and *in vitro* mouse BMMs. Sequencing libraries were constructed using the Illumina TruSeq RNA Sample Preparation Kit. Briefly, poly(A) mRNA was enriched, fragmented, and reverse-transcribed. Following adapter ligation and PCR amplification, library quality was verified via a Qubit Fluorometer (Thermo Fisher, USA) and an Agilent 2100 Bioanalyzer. Finally, paired-end sequencing (2×150 bp) was performed on an Illumina NovaSeq 6000 platform. This generated approximately 50 million raw reads per sample.

Raw sequencing data required rigorous quality control. First, we evaluated read quality using FastQC (v0.11.9). Next, fastp (v0.20.1) was applied to trim adapter sequences, low-quality bases (Q < 20), and reads under 50 bp. These filtered, high-quality reads were then aligned to reference genomes. Specifically, rat and mouse samples were mapped to the rn6 and mm10 genomes, respectively, using HISAT2 (v2.2.1) with default parameters.

Gene expression was quantified using StringTie (v2.1.5). Values were normalized and calculated as TPM and FPKM. To identify differentially expressed genes (DEGs), we utilized the DESeq2 package (v1.34.0) in R (v4.1.2). The edgeR package served as an auxiliary validation tool. Statistical significance was strictly defined. Genes with a false discovery rate (FDR) < 0.05 and |log2 fold change| > 1.0 were classified as DEGs. Their expression patterns were subsequently visualized using volcano plots and hierarchical clustering heatmaps.

Downstream analyses focused on functional interpretation. Gene Ontology (GO) enrichment was performed using clusterProfiler (v4.2.0). This analysis systematically categorized DEGs into biological processes, molecular functions, and cellular components. Additionally, we investigated Protein-Protein Interaction (PPI) networks. High-confidence interactions (score > 0.7) were extracted from the STRING database (v11.5). The resulting molecular network was then visualized utilizing Cytoscape (v3.9.0). All P-values were adjusted for multiple testing using the Benjamini-Hochberg method in R (v4.1.2).

### Isolation of primary BMMs and osteoclast induction

Primary bone marrow-derived macrophages (BMMs) were isolated from the femurs and tibias of 4-week-old C57BL6/J mice. Bone marrow cavities were flushed with α-MEM medium (1% FBS). Cells were cultured in α-MEM (10% FBS) for 3 days. Non-adherent cells were harvested and cultured with 30 ng/mL M-CSF (Novoprotein, CB34, China) for another 3 days to obtain adherent BMMs. To induce mature osteoclasts, BMMs were seeded into plates and cultured for 5 days in an induction medium containing 30 ng/mL M-CSF and 100 ng/mL RANKL (Novoprotein, CJ94, China). For mechanistic assays, cells were treated with recombinant BMP9 protein (MedChemExpress, HY-P70636, USA) and the BMP receptor inhibitor LDN-193189 (MedChemExpress, HY-12071, USA), or an adenovirus-mediated BMP9 vector (Adv-BMP9). This vector utilized a pBHGlox(Δ)E1,3Cre backbone driven by a CMV promoter. Viral transduction was strictly performed at a multiplicity of infection of approximately 250. Throughout all treatment and induction protocols, fresh culture media were replaced every 2 days.

### *In vitro* TRAP staining

Following 5 days of induction, BMM-derived osteoclasts were washed with PBS, fixed with 10% formaldehyde for 10 min, and stained using a commercial TRAP kit (Sigma, 378A, USA) at 37 °C for 15–20 min in the dark. TRAP-positive multinucleated cells (≥3 nuclei) were counted under a microscope. All cell culture assays were performed with at least three independent biological replicates (N≥3).

### Western blot

To extract total cellular proteins, cells were lysed on ice using RIPA buffer supplemented with protease and phosphatase inhibitors. Protein concentrations were determined via the BCA assay. Equal amounts of protein samples (20 μg) were adjusted to a uniform concentration using 5× SDS-PAGE loading buffer and denatured by boiling at 95 °C for 5 minutes. Proteins were separated by 10% SDS-PAGE and transferred onto PVDF membranes via the wet-transfer method. Membranes were blocked in 5% skimmed milk, incubated with primary antibodies (p-Smad1/5/9, GAPDH) overnight at 4 °C, washed with TBST, and incubated with HRP-labeled secondary antibodies for 1.5 hours at room temperature. Bands were visualized using an ECL substrate and quantified using ImageJ software, with GAPDH serving as the internal loading control.

### Statistical analysis

All data are expressed as the mean ± Standard Deviation (SD). All *in vitro* experiments (qPCR, TRAP staining, Western blot) were independently repeated at least three times (N≥3 biological replicates). Statistical analyses were performed using GraphPad Prism 10. Data normality was assessed by the Kolmogorov-Smirnov test, and variance equality by the F-test. For comparisons between two groups, Student’s t-test or the Mann-Whitney U test was applied as appropriate. For comparisons among three or more groups, a one-way ANOVA followed by Tukey’s *post hoc* test was utilized. Correlation analysis was conducted using Kendall’s rank correlation coefficient. A p < 0.05 was considered statistically significant.

## Result

### Critical-sized bone defects lead to the formation of atrophic non-union

**Figure 1:**
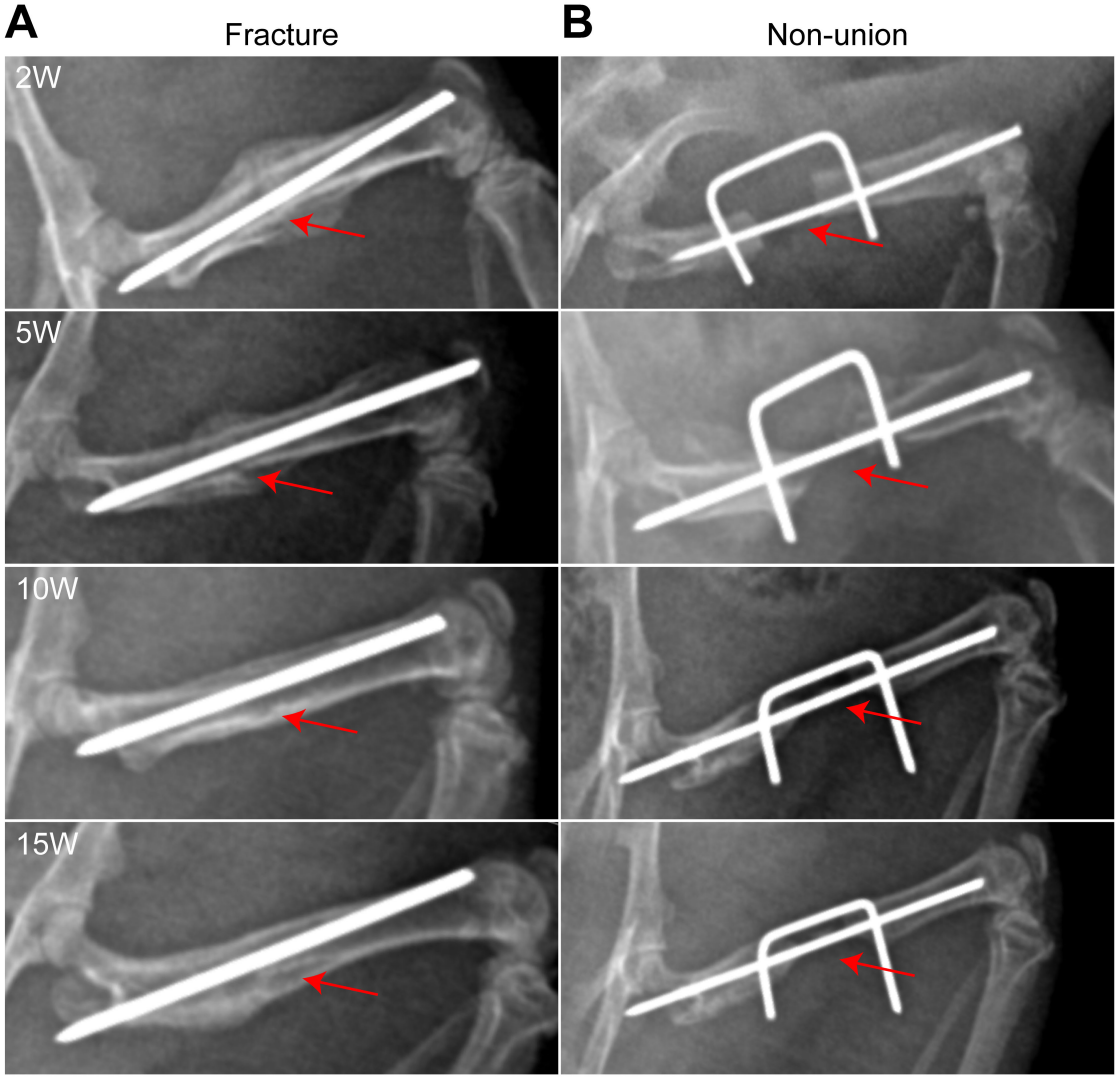
Radiographs of the fracture and non-union models at 2, 5, 10, and 15 weeks post-operation. **(A)** Representative radiographs of the femur of fracture group; **(B)** Representative radiographs of the femur of non-union group. Red arrows indicate the initial fracture or bone defect sites.

To evaluate fracture healing, radiographs were taken at 2, 5, 10, and 15 weeks postoperatively. In the fracture group, bone callus appeared around the fracture site at 2 weeks postoperatively. Within the bone callus, the original fracture line remained visible. By week 5, a continuous bony bridge had spanned the fracture gap. The fracture line became less distinct, and the volume of the external callus began to decrease. By weeks 10 and 15, cortical bone continuity was restored. As the medullary cavity gradually recanalized, the fracture line disappeared completely, confirming that bone healing was complete (**Figure 1A**). In contrast, the non-union group showed a failure to heal. Throughout the 15-week observation period, no radiographic evidence of mineralized bridging tissue was observed at the defect site. The gap between the bone ends persisted. Over time, the adjacent cortical margins underwent sclerosis, gradually forming narrowed, rounded bone ends (**Figure 1B**). These results confirm the successful establishment of an atrophic non-union model via critical-size defects.

### The non-union microenvironment shows aberrant intramembranous ossification and persistent tissue fibrosis

**Figure 2:**
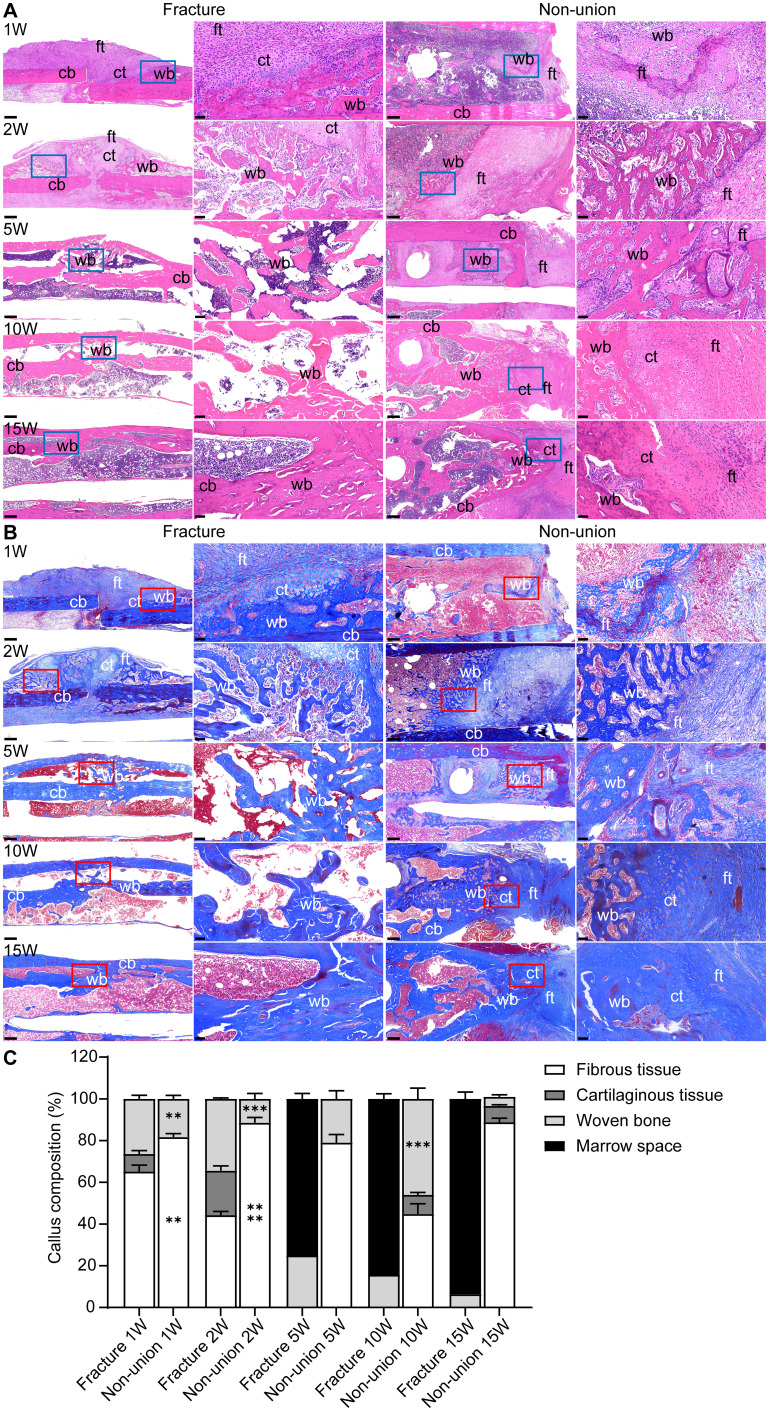
Histological and quantitative evaluation of the fracture and non-union models at 1, 2, 5, 10, and 15 weeks post-operation. **(A, B)** H&E staining and Masson’s trichrome staining in the callus regions. The image on the right shows a high-magnification view of the boxed area. **(C)** Quantitative analysis of callus composition. cb, cortical bone; wb, woven bone; ct, cartilaginous tissue; ft, fibrous tissue. Scale bars: 250 μm (left low magnification) and 50 μm (right high magnification). Data are presented as mean ± SD, N≥3. **P < 0.01, ***P < 0.001, Compared with the fracture group from the same period.

To investigate whether osteogenesis in non-union differs from fracture healing, histological evaluations were conducted using H&E and Masson’s trichrome staining at 1, 2, 5, 10, and 15 weeks postoperatively. In the fracture group, endochondral ossification was observed. At 1 and 2 weeks postoperatively, a soft callus formed around the fracture site, and woven bone appeared beneath the cartilage tissue. By week 5, the cartilage template had been resorbed and replaced by woven bone, forming a bridging hard bone callus. At weeks 10 and 15, remodeling restored the mature cortical bone structure. The medullary cavity was reestablished, indicating complete histological healing (**Figures 2A, B**). In the non-union group, the defect gap was filled with fibrous tissue at weeks 1 and 2. The formation of woven bone was limited to the margins of the fibrous tissue, and no chondrocytes were observed. This indicates that intramembranous ossification occurred in the non-union site. By week 5, fibrous tissue was still present, and no bone bridge had formed at the defect site. However, woven bone had begun to obliterate the medullary cavity at the bone end. At weeks 10 and 15, no bony bridge had yet formed; the bone ends were enclosed by cortical bone, and a typical cartilaginous cap had formed. These histological changes confirmed the development of atrophic non-union.

We then quantified the tissue components in the callus. The results showed that, between 1 and 2 weeks, the percentage of fibrous tissue in the callus of the non-union group was significantly higher than that in the fracture group, while the percentage of woven bone was significantly lower. Starting at 5 weeks postoperatively, fibrous and cartilaginous tissues were absent in the fracture group, and bone bridging formed; in contrast, the non-union group consistently maintained a high percentage of fibrous tissue (**Figure 2C**). These findings align with the radiological results and further confirm the formation of atrophic non-union.

### Transcriptomics reveals the 1–2 week post-fracture window dictates non-union development

**Figure 3:**
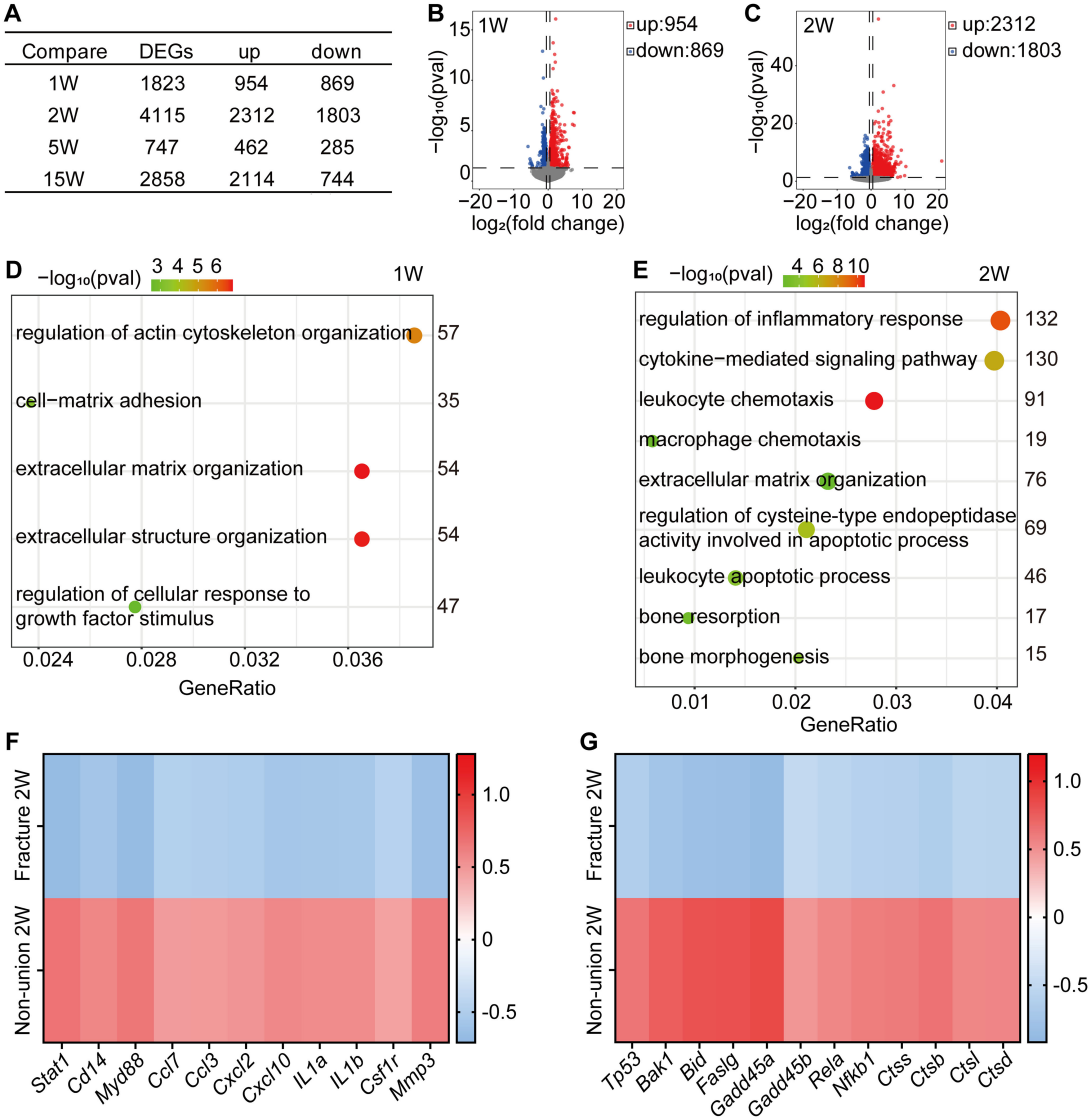
Transcriptomic analysis confirms significant upregulation of inflammation and apoptosis in the early stages of non-union. **(A)** Statistics table of differentially expressed genes; **(B, C)** Volcano plot of DEGs at 1 week and 2 weeks; **(D, E)** GO enrichment analysis of DEGs at 1 week and 2 weeks; **(F, G)** Heatmap of the expression of inflammation-related and apoptosis-related genes (Z-score, N = 3).

To elucidate the molecular differences between fracture healing and non-union, we performed RNA sequencing on tissue from the defect margins at 1, 2, 5, and 15 weeks postoperatively. The results showed that there were 1823 DEGs between the two groups at 1 week postoperatively and 4115 DEGs at 2 weeks post-surgery. However, at 5 and 15 weeks post-surgery, the number of DEGs between the two groups was lower than during the 1-2-week period (**Figures 3A, B**). Combined with histological changes, these results suggest that the 1–2 weeks post-surgery represent a critical window determining whether non-union will occur.

Then we used GO enrichment analysis to assess transcriptomic differences between the two groups. The results showed that, at week 1, DEGs in the non-union group were primarily enriched in pathways such as “extracellular matrix organization,” while no significant enrichment was observed in inflammation- and apoptosis-related pathways (**Figure 3D**). At 2 weeks postoperatively, DEGs were primarily enriched in inflammation-related pathways such as “regulation of inflammatory response” and “cytokine-mediated signaling,” as well as apoptosis-related pathways such as “positive regulation of the execution phase of apoptosis”. Additionally, pathways like “bone morphogenesis” and “bone resorption” were also significantly enriched (**Figure 3E**). Therefore, we generated heatmaps of the expression of inflammation- and apoptosis-related genes. We found that the expression of inflammation- and chemotaxis-related genes (e.g., *IL1a*, *Cxcl2*, *Myd88*) was significantly upregulated in the non-union group (**Figure 3F**). Furthermore, the expression of pro-apoptotic markers (e.g., *Tp53*) and cathepsins (e.g., *Ctss*) was significantly increased (**Figure 3G**). In summary, these results indicate significant early changes in the non-union microenvironment, including persistent inflammation, excessive apoptosis activation, and abnormal bone metabolism.

### The canonical BMP9-SMAD signaling axis is markedly suppressed during the early stage of non-union formation

**Figure 4:**
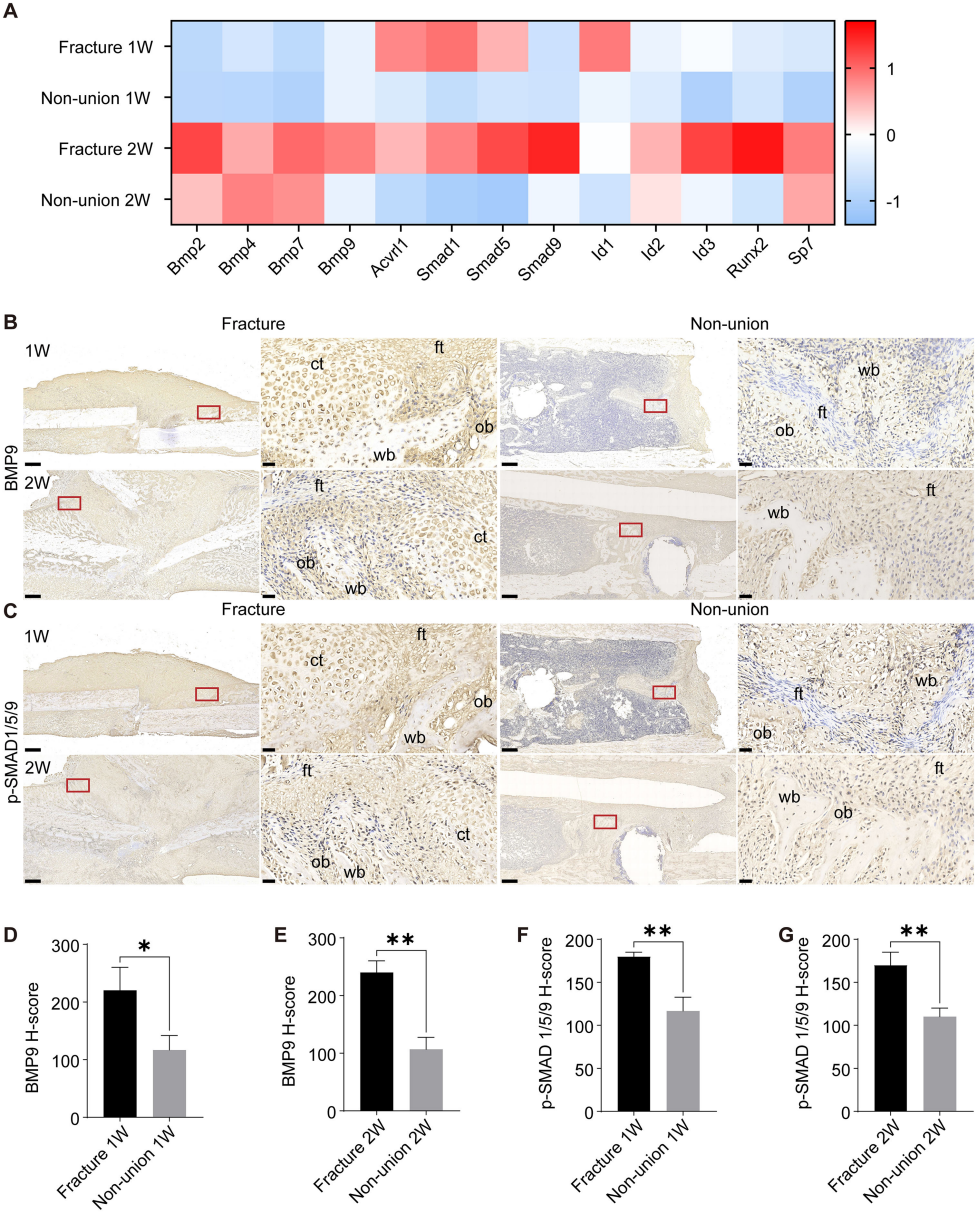
Impaired early BMP9 expression and canonical SMAD signaling activation within the non-union microenvironment. **(A)** Heatmap of BMP-SMAD pathway and bone formation-related gene expression at 1 and 2 weeks post-surgery (Z-score); **(B, C)** Representative immunohistochemistry images of BMP9 and p-SMAD1/5/9; **(D–G)** H-Score semi-quantification analysis of BMP9 and p-SMAD1/5/9 immunohistochemistry in the callus regions at 1 and 2 weeks post-surgery. cb, cortical bone; ob, osteoblast; wb, woven bone; ct, cartilaginous tissue; ft, fibrous tissue. Scale bars: 250 μm (left low magnification) and 50 μm (right high magnification). Data are presented as mean ± SD, N≥3. *P < 0.05, **P < 0.01.

Given the abnormal bone metabolism identified via GO enrichment analysis and the profound impact of the BMP/SMAD pathway on osteochondral morphogenesis, we evaluated the expression of BMP/SMAD signaling-related genes. The results showed no significant differences in the mRNA expression of Bmp2, Bmp4, Bmp7, and Bmp9 between the two groups at 1 week postoperatively. However, at 2 weeks, the canonical BMP/SMAD pathway was activated in the fracture group, and the transcriptional levels of key ligands (e.g., *Bmp9*), receptors (*Acvrl1*), intracellular transducers (e.g., *Smad1*, *Smad5*, *Smad9*), and downstream osteogenic targets (e.g., *Runx2*, *Sp7*) were significantly upregulated. In contrast, this transcriptional upregulation was not observed in the non-union group. Specifically, compared with the fracture group, the expression of *Bmp9* was significantly downregulated (log2 FC = -1.612), and the expression of downstream receptors and transcription factors was also significantly suppressed. These findings suggest that the early deficiency of *Bmp9* expression is a major contributing factor to the development of bone non-union (**Figure 4A**).

To validate these findings at the protein level, we evaluated the protein expression of BMP9 and p-SMAD1/5/9 *in situ* via IHC. The results showed that there was strong positive BMP9 expression in chondrocytes, osteoblasts and newly formed woven bone regions in the fracture group. Nuclear p-SMAD1/5/9 also exhibited a similar spatial distribution. In the non-union group, BMP9 was weakly positive in osteoblasts and woven bone regions, and the nuclear expression of p-SMAD1/5/9 was markedly attenuated compared to the fracture group (**Figures 4B, C**). This difference was further confirmed by semi-quantitative H-score analysis. At both time points postoperatively, the H-scores of BMP9 and p-SMAD1/5/9 in the non-union group were significantly lower than those in the fracture group (**Figures 4D–G**). These results demonstrate that the activation of the canonical BMP9-SMAD signaling axis is significantly suppressed during the early stages of bone non-union.

### Early non-union microenvironment induces deficient osteogenesis and hyperactive bone resorption

**Figure 5:**
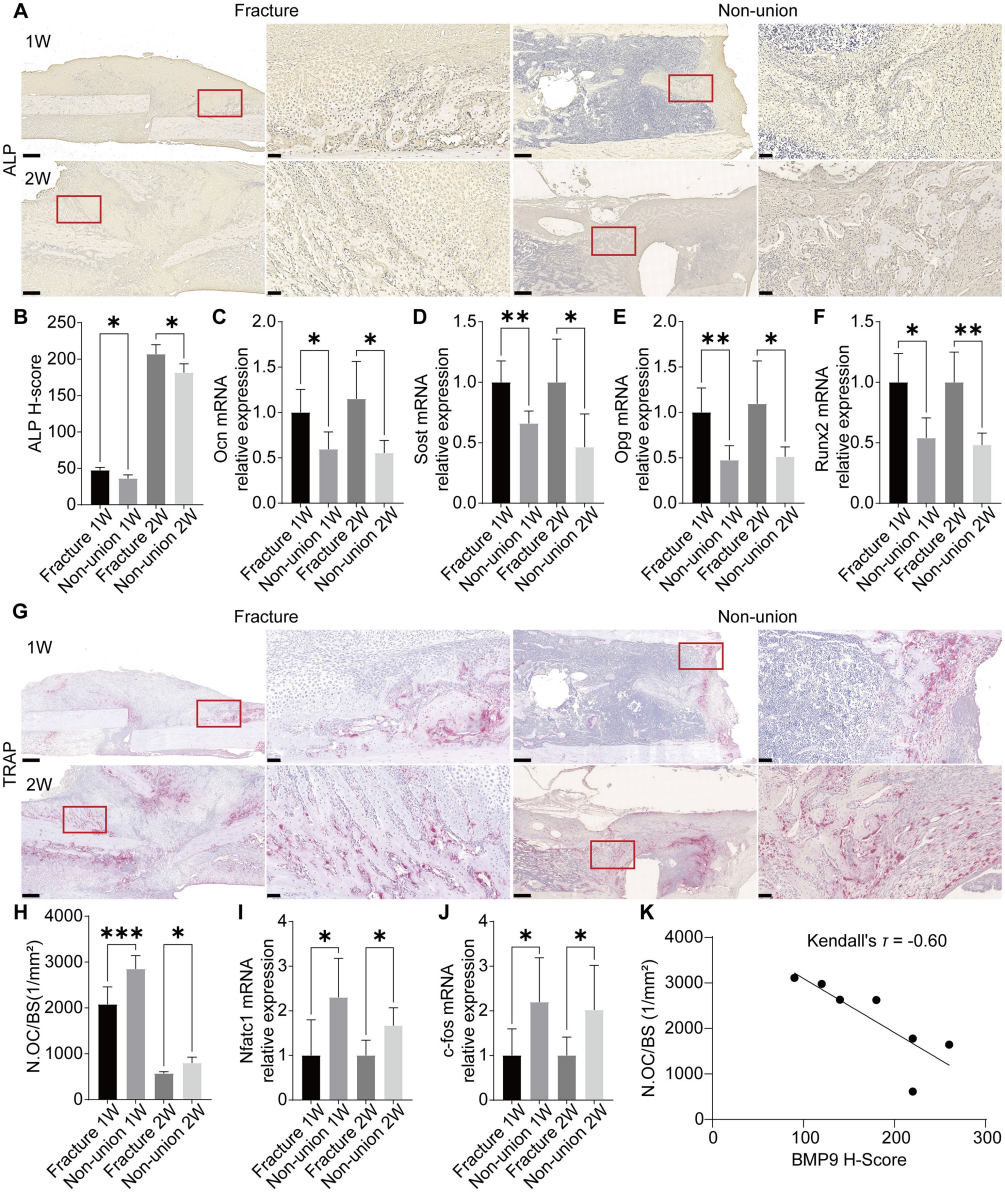
Impaired osteogenesis and enhanced bone resorption in early non-union correlate with abnormal BMP/SMAD signaling. **(A)** Representative immunohistochemistry images of ALP in the callus regions at 1 and 2 weeks post-operation; **(B)** H-score semi-quantitative analysis of ALP expression; **(C–F)** Relative mRNA expression levels of osteogenic markers (*Ocn*, *Sost*, *Opg*, *Runx2*); **(G)** Representative TRAP staining images of the callus regions at 1 and 2 weeks; **(H)** Quantitative analysis of osteoclast number per bone surface (N.Oc/BS); **(I, J)** Relative mRNA expression levels of osteoclastogenic markers (*Nfatc1, c-fos*); **(K)** Kendall’s rank correlation analysis between BMP9 H-score and N.Oc/BS. Scale bars: 250 μm (left low magnification) and 50 μm (right high magnification). Data are presented as mean ± SD, N≥3. *P < 0.05, **P < 0.01, ***P < 0.001.

To investigate the downstream cellular consequences of BMP9/SMAD signaling defects, and combined with the upregulation of “bone resorption” pathways identified via GO enrichment analysis, we evaluated the uncoupled bone remodeling within the non-union group. Initially, osteoblast activity was assessed via ALP staining. At 1 and 2 weeks postoperatively, the fracture group exhibited pronounced osteoblastic activity, demonstrating intense positive staining within both chondrocytes and newly formed woven bone regions. In contrast, the non-union group displayed a marked depletion of ALP-positive osteoblasts accompanied by significantly attenuated staining intensity (**Figure 5A**). Semi-quantitative H-score analysis confirmed this significant decrease in ALP expression (**Figure 5B**). Furthermore, we quantified the expression of osteogenic markers. The mRNA expression of key genes, including *Ocn*, *Opg*, *Sost*, and *Runx2*, was significantly decreased in the non-union group compared to the fracture group (**Figures 5C–F**).

Subsequently, we evaluated osteoclasts using TRAP staining. At 1 and 2 weeks postoperatively, the fracture group showed scattered TRAP-positive signals in the woven bone areas. In contrast, continuous TRAP-positive signals were observed in the woven bone areas of the non-union group. Furthermore, the TRAP-positive cells attached to the bone surface exhibited a larger volume and more nuclei (**Figure 5G**). Quantitative analysis confirmed that the number of osteoclasts was significantly higher in the non-union group (**Figure 5H**). Correspondingly, the mRNA expression of osteoclast formation markers, such as *Nfatc1* and *c-fos*, was significantly increased in the non-union group (**Figures 5I, J**). To clarify whether this change in osteoclasts is related to defects in BMP9/SMAD signaling, we conducted a correlation analysis between the two, which revealed a strong negative correlation (Kendall’s *τ* = -0.60) (**Figure 5K**). These results suggest that the absence of BMP9/SMAD signaling in the non-union group is one of the reasons for the abnormal increase in bone resorption.

### BMP9 suppresses RANKL induced osteoclastogenesis via the canonical SMAD pathway *in vitro*

**Figure 6:**
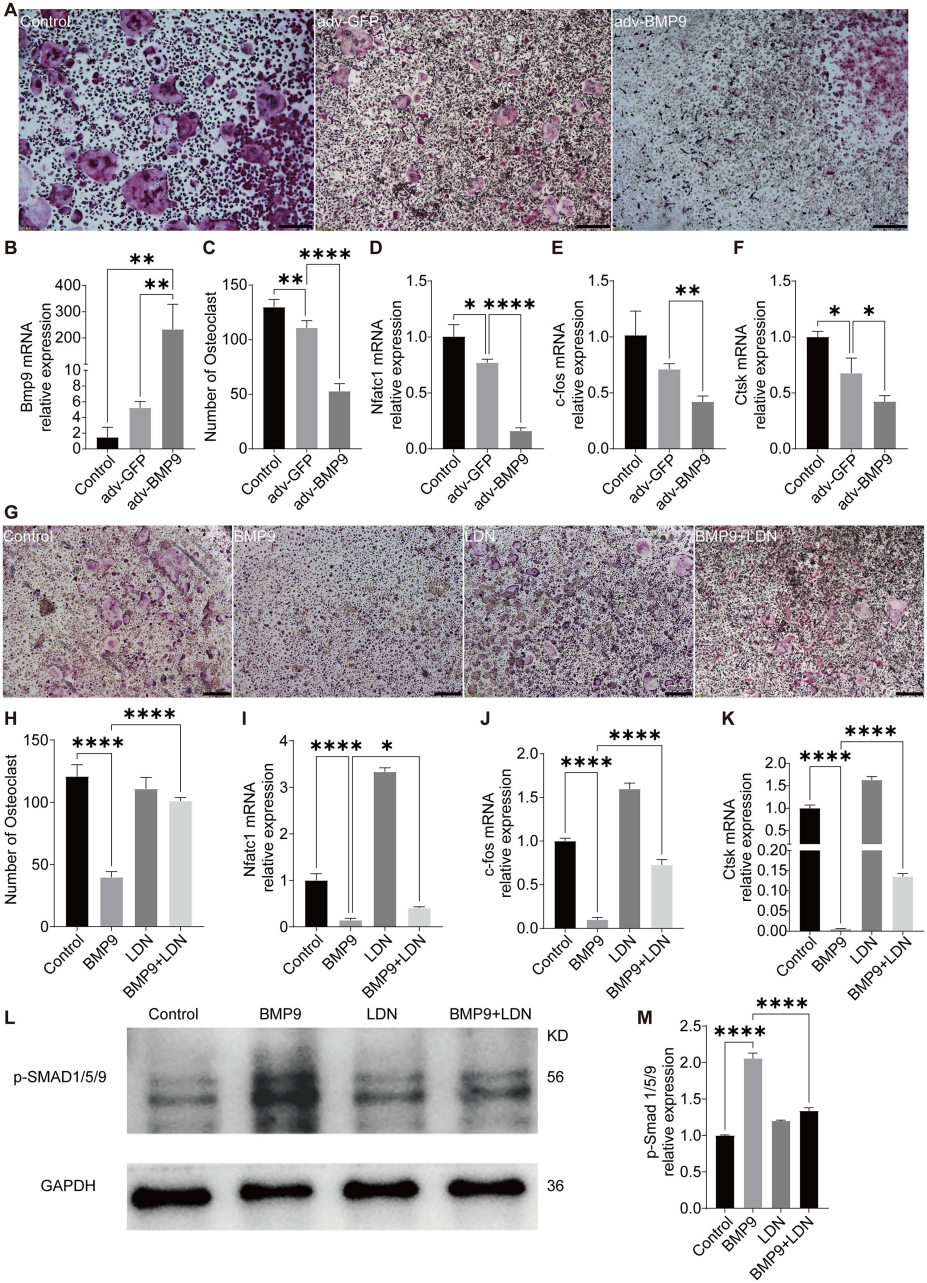
BMP9 suppresses osteoclastogenesis *in vitro* via the canonical SMAD signaling pathway. **(A)** Representative TRAP staining images of BMMs in the uninfected control (Control), empty vector (adv-GFP), and BMP9-overexpressing (adv-BMP9) groups. **(B)** RT-qPCR validation of *Bmp9* overexpression; **(C)** Quantitative analysis of TRAP-positive multinucleated osteoclasts; **(D–F)** Relative mRNA expression levels of osteoclastogenic markers (*Nfatc1, c-fos, Ctsk*); **(G)** Representative TRAP staining images of BMMs treated with the blank control, recombinant BMP9, LDN-193189, or their combination; **(H)** Quantitative analysis of TRAP-positive multinucleated osteoclasts; **(I–K)** Relative mRNA expression levels of osteoclastogenic markers (*Nfatc1, c-fos, Ctsk*); **(L, M)** Representative Western blot images of p-SMAD1/5/9 and GAPDH, along with the corresponding quantitative analysis. Scale bars: 200 μm. Data are presented as mean ± SD, N≥3. *P < 0.05, **P < 0.01, ****P < 0.0001.

To determine whether BMP9 has a regulatory effect on osteoclastogenesis, we evaluated this mechanism using primary mouse bone marrow-derived macrophages (BMMs). We first investigated this using adenovirus-mediated BMP9 (adv-BMP9) and found that, compared to cells infected with the empty vector(adv-GFP), cells overexpressing BMP9 had significantly fewer RANKL-induced osteoclasts and exhibited inhibited differentiation (**Figures 6A–C**). Correspondingly, the mRNA expression of osteoclast differentiation markers *Nfatc1* and *c-fos*, and the bone resorption marker *Ctsk*, was significantly reduced.

However, it is worth noting that infection with Adv-GFP also affected the differentiation of osteoclasts. Therefore, we further investigated the regulatory effect of BMP9 on osteoclasts using recombinant BMP9 and the specific ALK2/3 receptor inhibitor LDN-193189 (LDN). Consistent with the previous results, recombinant BMP9 significantly inhibited the differentiation of osteoclasts. Crucially, the use of LDN to inhibit the canonical BMP/SMAD signaling significantly reversed the inhibitory effect of BMP9 on osteoclastogenesis (**Figures 6G, H**) and restored the expression of *Nfatc1*, *c-fos*, and *Ctsk* (**Figures 6I–K**). Western blot results further confirmed that BMP9 significantly upregulated the expression of p-SMAD1/5/9, while LDN significantly blocked the activation of this canonical pathway (**Figures 6L, M**). These results confirm that BMP9 inhibits osteoclast differentiation and function through the canonical SMAD signaling pathway.

### BMP9 regulate the early chemokines *Ccl2* and *Ccl7* to promote pro-reparative M2 macrophage polarization

**Figure 7:**
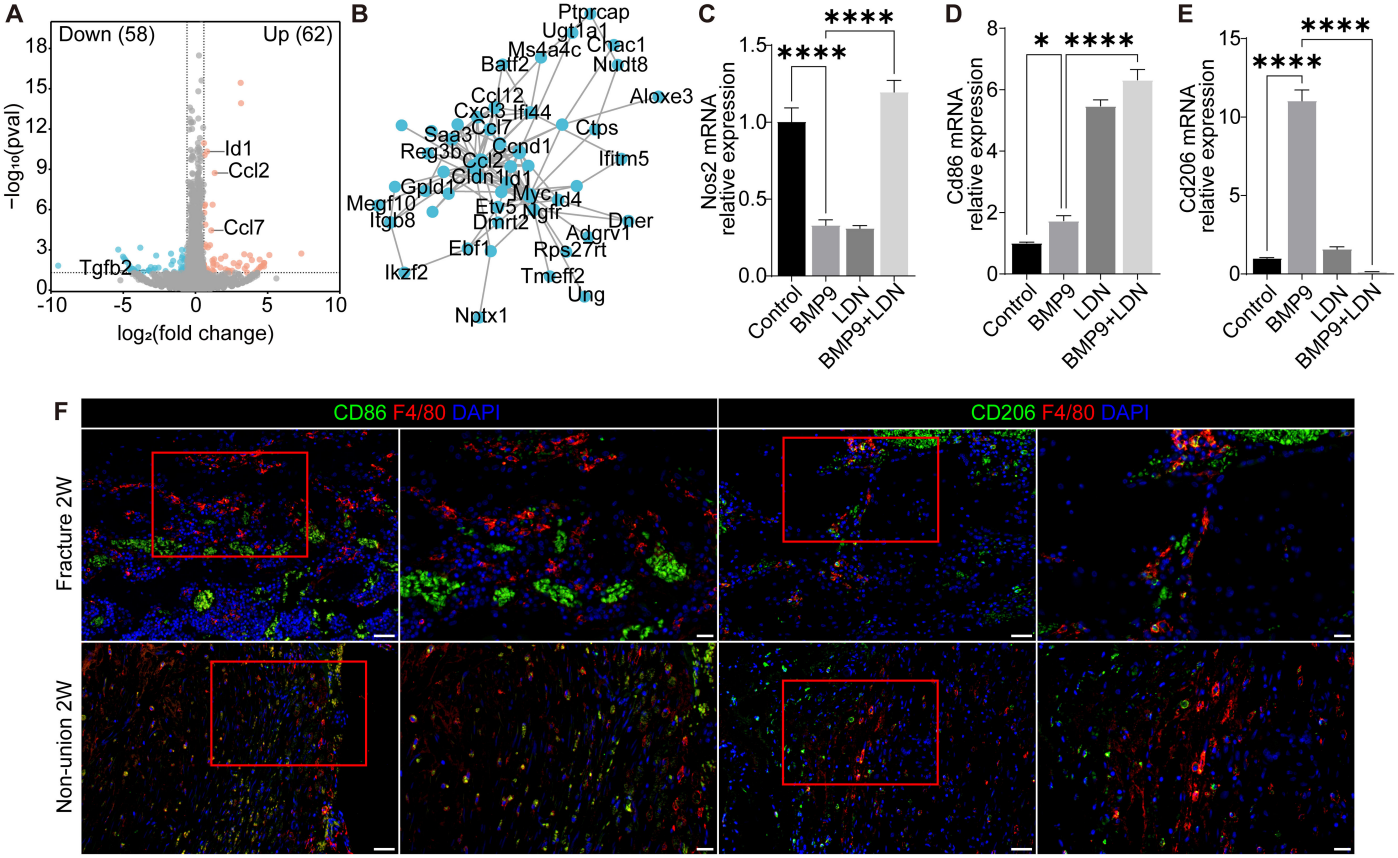
BMP9 regulates macrophage polarization via the canonical SMAD pathway to modulate the local inflammatory response. **(A)** Volcano plot showing DEGs, from RNA-seq analysis comparing BMP9-treated BMMs to untreated controls; **(B)** Protein-protein interaction (PPI) network of the DEGs, highlighting genes associated with macrophage chemotaxis and polarization; **(C–E)** Relative mRNA expression levels of M1 markers (*Nos2*, *Cd86*) and M2 marker (*Cd206*) in BMMs treated with recombinant BMP9, LDN-193189, or their combination; **(F)** Representative immunofluorescence images of M1 macrophages (CD86, green) or M2 macrophages (CD206, green) with the pan-macrophage marker F4/80 (red) in the callus regions of the fracture and non-union groups at 2 weeks post-operation. Nuclei were counterstained with DAPI (blue). The image on the right shows a high-magnification view of the boxed area. Scale bars: 50 μm (left low magnification) and 20 μm (right high magnification). Data are presented as mean ± SD, N≥3. *P < 0.05, ****P < 0.0001.

Given that osteoclasts and macrophages share a common myeloid progenitor, and transcriptomic analysis indicated abnormal inflammatory activation in the non-union group, we further investigated whether BMP9 impacts local immunity. Transcriptional analysis of BMP9-treated BMMs revealed an increase in *Id1* expression, further confirming the activation of the canonical SMAD signaling pathway. More importantly, we observed a significant upregulation in the expression of early chemokines, specifically *Ccl2* and *Ccl7* (**Figure 7A**). Furthermore, PPI analysis revealed that CCL2 and CCL7 are key nodes interacting directly with the TGF-β/BMP signaling hub (**Figure 7B**).

Because CCL2 and CCL7 are typically associated with macrophage polarization, we evaluated the expression of M1 polarization markers (e.g., *Nos2*, *Cd86*) and the M2 polarization marker (e.g., *Mrc1*). We found that the expression of *Nos2* and *Cd86* was significantly reduced following BMP9 treatment, while the expression of *Mrc1* was significantly increased. Crucially, LDN treatment effectively abrogated the regulatory effect of BMP9 on macrophage polarization (**Figures 7C–E**).

To clarify whether this regulatory process is mechanistically linked to the abnormal inflammation observed in the non-union group, we performed immunofluorescence staining on tissues at 2 weeks postoperatively. The standard fracture group exhibited significant infiltration of both M1 macrophages (CD86^+^) and M2 macrophages (CD206^+^). However, in the non-union group, CD206^+^ M2 macrophages were almost entirely depleted, while CD86^+^ M1 macrophages showed a significant increase and dense cellular aggregation. These results indicate that the early deficiency of BMP9/SMAD signaling significantly disrupts the expression of chemokines and impairs macrophage polarization towards the M2 phenotype, leading to sustained inflammatory infiltration.

## Discussion

The pathophysiological mechanisms driving atrophic non-union have historically been attributed to late-stage mechanical instability or deficient revascularization (2). However, emerging perspectives in osteoimmunology propose that the definitive outcome of skeletal repair is largely established during the initial inflammatory phase (9). Our study utilized a 6-mm critical-size defect (CSD) rat model. While this structural void surpasses the inherent biological capacity for spontaneous bridging, it serves as a reliable experimental platform to isolate the early biological impediments to healing. Specifically, these include a failure to resolve early inflammation and an inability to initiate endochondral ossification (18, 24). Temporal transcriptomic and histological profiling suggests that the non-union group exhibited persistent inflammation and fibrosis, rather than an effective osteogenic process during the critical 1–2 weeks post-fracture window.

An unexpected finding in our study is the temporal discordance between the absence of local *Bmp9* transcripts at week 1 and the positive BMP9 protein expression detected via immunohistochemistry. BMP9 is a circulating endocrine factor predominantly synthesized by the liver (25). Given this systemic profile, we hypothesize that the BMP9 protein present within the acute hematoma originates from vascular extravasation rather than local transcription. It must be explicitly stated, however, that this mechanism was not directly tested in the current study and remains a hypothesis requiring future experimental validation. As normal healing progresses to week 2, the osteogenic differentiation of mesenchymal progenitors is accompanied by *de novo* local transcription of *Bmp9* (26). In contrast, this localized transcriptional activation is deficient within the non-union microenvironment. Consequently, the tissue suffers a deficiency in the BMP9-SMAD signaling axis exactly during the critical temporal window when it is poised to transition into the reparative phase (27).

Our findings also address the ongoing debate regarding the direct regulatory role of BMP9 in osteoclastogenesis. While previous *in vitro* studies utilizing immortalized macrophage lines suggested that BMP9 might promote osteoclast differentiation (21), our data demonstrate that in primary BMMs, BMP9 acts as a significant negative regulator of RANKL-induced osteoclastogenesis. This is achieved through the canonical SMAD pathway. These findings are consistent with the *in vivo* research results of Zhou et al. (22). Mechanistically, BMP9 upregulates ID1, a dominant-negative helix-loop-helix (HLH) protein. ID1 interacts with basic HLH transcription factors such as MITF, thereby preventing the transactivation of the Nfatc1 promoter. By suppressing this key osteoclastogenic program, the canonical BMP9-SMAD-ID1 axis serves to protect the early callus from excessive, pathological resorption (28–30).

Furthermore, a successful healing cascade demands a coordinated transition from pro-inflammatory (M1) to pro-reparative (M2) macrophage phenotypes (31). Transcriptomic analysis of BMP9-treated BMMs revealed a significant upregulation of the chemokines *Ccl2* and *Ccl7*. When evaluating this altered chemokine profile, the potential distinction between standard inflammatory cell infiltration and physiological progenitor recruitment warrants consideration. While the CCL2/CCL7-CCR2 signaling axis is classically characterized as a driver of destructive inflammation, its biological output is highly microenvironment-dependent (32). We propose that BMP9 may recontextualize the biological output of these chemokines. Rather than fueling an uncoordinated pro-inflammatory influx, BMP9-induced CCL2 and CCL7 likely act as targeted homing signals. They recruit essential circulating monocytes and mesenchymal progenitors directly to the fracture hematoma (32). Crucially, the continuous local presence of BMP9 appears to facilitate the polarization of these newly arrived myeloid cells toward a pro-reparative M2 phenotype upon arrival (33). In the non-union microenvironment, the deficiency of BMP9 signaling disrupts this critical recruitment and polarization function. Consequently, the recruited myeloid cells tend to remain in a chronic M1-dominant profile. This dysregulated microenvironment is histologically associated with persistent inflammatory infiltration and subsequent tissue fibrosis.

It is essential to contextualize these findings within a broader clinical framework. While our isolated injury model enabled a controlled investigation into the local BMP9-driven osteoimmune axis, the clinical management of severe, high-energy fractures frequently occurs within the complex landscape of polytrauma. Systemic inflammatory derangements, such as the cytokine storms associated with multiple extremity injuries, may disrupt local inflammatory responses identified in our model (34). Indeed, recent studies have shown that multisystem limb trauma can alter macrophage polarization, and treatment strategies that promote macrophage polarization towards the M2 phenotype can mitigate systemic inflammation and abnormal osteogenesis (35).

Finally, we acknowledge two primary limitations of our study. First, an *in vivo* rescue experiment utilizing local delivery of recombinant human BMP9 (rhBMP9) was not performed. Nevertheless, classic studies by He and colleagues have established BMP9 as a potent osteogenic factor capable of promoting bone formation across various defect models (36, 37). Building upon their foundational work, our findings suggest that targeted modulation of the BMP9-driven bone-immune axis warrants further investigation as a therapeutic strategy. While clinically approved options like rhBMP-2 are highly osteoinductive, they are frequently limited by complications such as exaggerated osteoclast activation and premature graft resorption. Our data suggest that BMP9, by simultaneously supporting osteogenesis and limiting osteoclastogenesis via immune modulation, may provide a potential solution for early intervention in bone non-union. Future research should adopt interdisciplinary immune regeneration strategies to achieve controlled release delivery and safely utilize BMP9 driven bone immune axis (38). Secondly, although current research strictly focuses on this local microenvironment, complex neuroimmune crosstalk, such as the effects of early peripheral nerve reinnervation and systemic neuroendocrine stress response on macrophage polarization, deserves further investigation as potential upstream regulators of this signaling axis (39).

## Data Availability

The datasets presented in this study can be found in online repositories. The names of the repository/repositories and accession number(s) can be found below: https://figshare.com/, https://figshare.com/s/e1564196fd5c46a6b5a9.
